# The Mississippi State University College of Veterinary Medicine Shelter Program

**DOI:** 10.3390/ani5020259

**Published:** 2015-04-28

**Authors:** Philip Bushby, Kimberly Woodruff, Jake Shivley

**Affiliations:** College of Veterinary Medicine, Mississippi State University, P.O. Box 6001, Mississippi State, MS 39762, USA; E-Mails: kwoodruff@cvm.msstate.edu (K.W.); jshivley@cvm.msstate.edu (J.S.)

**Keywords:** Surgical instruction, animal shelter, shelter medicine, spay/neuter, shelter education, shelter management, veterinary education

## Abstract

**Simple Summary:**

First initiated in 1995 to provide veterinary students with spay/neuter experience, the shelter program at the Mississippi State University College of Veterinary Medicine has grown to be comprehensive in nature incorporating spay/neuter, basic wellness care, diagnostics, medical management, disease control, shelter management and biosecurity. Junior veterinary students spend five days in shelters; senior veterinary students spend 2-weeks visiting shelters in mobile veterinary units. The program has three primary components: spay/neuter, shelter medical days and Animals in Focus. Student gain significant hands-on experience and evaluations of the program by students are overwhelmingly positive.

**Abstract:**

The shelter program at the Mississippi State University College of Veterinary Medicine provides veterinary students with extensive experience in shelter animal care including spay/neuter, basic wellness care, diagnostics, medical management, disease control, shelter management and biosecurity. Students spend five days at shelters in the junior year of the curriculum and two weeks working on mobile veterinary units in their senior year. The program helps meet accreditation standards of the American Veterinary Medical Association’s Council on Education that require students to have hands-on experience and is in keeping with recommendations from the North American Veterinary Medical Education Consortium. The program responds, in part, to the challenge from the Pew Study on Future Directions for Veterinary Medicine that argued that veterinary students do not graduate with the level of knowledge and skills that is commensurate with the number of years of professional education.

## 1. Introduction

The American Veterinary Medical Association (AVMA) Council on Education (COE) requires veterinary schools to “provide instruction in both the theory and practice of medicine and surgery.” The accreditation standards for veterinary colleges state that “must include principles and hands-on experiences in physical and laboratory diagnostic methods and interpretation, … disease prevention, biosecurity, therapeutic intervention (including surgery)” among several other skills [1] The recent study by the North American Veterinary Medical Educational Consortium (NAVMEC) concluded “to achieve entry-level competency, students should be provided sufficient time and opportunity to learn and practice necessary knowledge and skills [2].” It is, therefore, clear that one of the expectations of a veterinary curriculum is to provide students with ample hands-on practical experience. Yet the Pew National Veterinary Education Program’s study entitled Future Directions for Veterinary Medicine concluded, in part, that “veterinary graduates, with few exceptions, have been unable to acquire the level of knowledge and skills which should be achieved, considering the many years devoted to a professional veterinary education [3].”

In 1995, in an effort to increase the hands-on clinical experience of veterinary students the College of Veterinary Medicine at Mississippi State University developed a cooperative program with a local animal shelter. Over the years the shelter program has grown in stages. Early on the program focused on spay/neuter surgery. From 1995 to 2007 the program was limited to one or two local shelters that had their own surgical suite. In 2007, the College obtained a mobile veterinary unit. No longer limited to shelters with a surgical suite the program rapidly grew to serve 14 different animal shelters. In 2013, with the addition of a second mobile veterinary unit the program expanded to 20 animal shelters and enrollment was opened as an extern experience for students from other veterinary schools. In 2014 the program was expanded to include an increased emphasis on shelter animal wellness care, medical management and shelter management as well as developing an educational component that targets elementary school children.

## 2. Program Development

### 2.1. The Early Years, 1995 to 2007

During the early years the shelter program at the Mississippi State University College of Veterinary Medicine involved just third year veterinary students. Third year students have nine months of clinical activities in the veterinary teaching hospital. This includes a 6-week primary care rotation focused on routine small animal care. Initially every student in the primary care rotation spent one day at a local animal shelter performing spays and neuters under the supervision of a board certified surgeon. Students took turns rotating though the surgical suite performing surgeries with a faculty surgeon scrubbed in as their assistant. The role of the faculty member was to guide the student through the procedures, intervene to prevent errors, help the students when there were problems, and provide verbal and written feedback to the students after the surgeries were completed.

Conducted as a “mash style” spay/neuter activity, all necessary surgical supplies were transported to the shelter along with the students and faculty member. The shelter provided the surgical suite, surgical table, anesthetic machine and, of course, the animals. Shelter personnel would select the animals they wished to present for surgery. Pending the results of a brief physical examination, patients would be anesthetized, prepped for surgery and transported to the surgical suite one at a time. Cases were assigned to give each student equal opportunity to perform each of the surgical procedures; cat spay, cat castration, dog spay, and dog castration.

Initially all surgeries were performed on adult (over 6 months old) dogs and cats. Shelter management resisted the practice of early age or pediatric spay/neuter in spite of the fact that a majority of adoptions were puppies and kittens. Eventually, with a change in shelter management, pediatric spay/neuter was added to the program thereby providing students with experience in performing spays and neuters of puppies and kittens as young as six to eight weeks of age.

In 2005 the shelter program doubled in size. When a local humane society built a new animal shelter that included a surgical suite, the college expanded the program to include that shelter. Rather than reduce the trips to the original shelter the approach was to simply add more trips. With this addition every third year student spent 2 days (one in each of the two shelters) performing spays and neuters.

### 2.2. Mobile Veterinary Units

**Figure 1:**
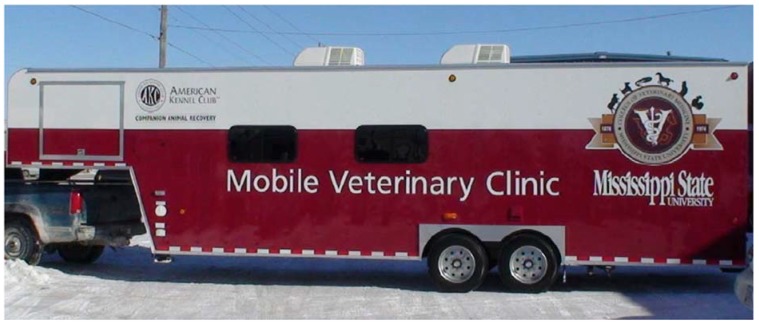
Mississippi State University College of Veterinary Medicine’s first mobile unit. Mississippi State University College of Veterinary Medicine’s first mobile unit is a 32 ft gooseneck trailer fully equipped for spay/neuter of dogs and cats at animal shelters

The College obtained its first mobile veterinary unit as a result of Hurricane Katrina. In 2005, Hurricane Katrina created massive destruction on the Mississippi and Louisiana gulf coast. One of the aftermaths of Hurricane Katrina was an increase in funding for animal rescue and spay/neuter along the gulf coast. A grant from the American Kennel Club Companion Area Recovery along with private donations allowed for the purchase of a mobile veterinary unit designed for disaster recovery and spay/neuter (See Figure 1). A grant from the Humane Society of the United States provided funding to expand the shelter program and operate the mobile unit.

With the acquisition of the mobile unit the college was no longer limited to shelters that had their own surgical suite, so the program rapidly expanded to 14 shelters. The college created a senior year shelter medicine spay/neuter elective rotation. The 2-week rotation functioned year round except for Christmas and New Year’s break and could enroll two senior students at a time. Shelters would still select their patients, but with the mobile unit the program was no longer limited to just one surgery being performed at a time. The junior students still made two trips to shelters and still scrubbed in with a faculty member acting as assistant. Junior students averaged 15 sterilization surgeries in those two trips. Up to 50 senior students could enroll in the elective each year. Senior students would make seven or eight trips to shelters. On the first day of the 2-week elective a faculty member would scrub in with the senior students for each surgery, but after that first day senior students performed the surgeries unassisted. A faculty member or resident was always supervising surgeries and could scrub in to assist if necessary, but for the most part students performed the surgeries on their own. As long as the safety of the patient was not at risk, students were given the chance to handle problems/complications on their own. Senior students would average over 70 surgeries each during the 2-week rotation. With this many surgeries in a concentrated period of time, most students became efficient and confident in their surgical skills.

**Figure 2:**
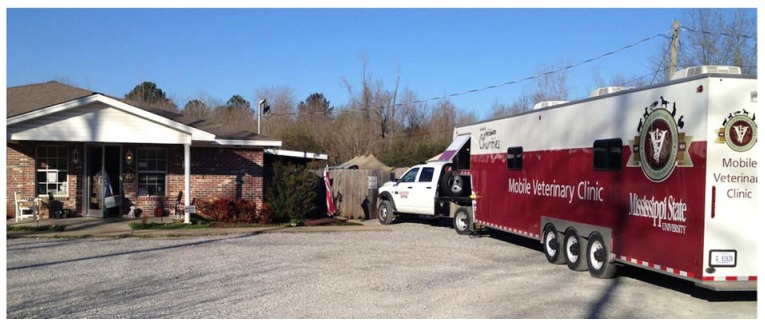
Mississippi State University College of Veterinary Medicine’s second mobile unit at a local animal shelter. Mississippi State University College of Veterinary Medicine’s second mobile unit is a 38 ft gooseneck trailer equipped with three surgical tables for performing spays and neuters

The program still had limited enrollment during this period. Enrolling two students for 2-weeks at a time, 50 weeks of the year, kept enrollment at 50 students a year. Demand for the elective was great but with a class size of 80 to 85 students per year approximately 40% of each senior class was unable to enroll. Discontented with the situation, the freshmen Class of 2014 initiated a project to raise funds to purchase a second mobile unit. When PetSmart Charities heard of the students efforts they funded a grant to purchase a second mobile unit (See Figure 2).

The second mobile unit was put into service in January of 2013, just in time for the Class of 2014 to be the first class in which every student who wished to take the Shelter elective could enroll. Every junior student still goes on two trips, but the capacity for senior students expanded from 50 to 120. With an enrollment of 85 students per class at Mississippi State University College of Veterinary Medicine there is room to enroll between 35 and 40 senior students from other schools in the elective. The 2-week elective is offered 48 weeks per year and can accommodate five senior students each rotation. Each mobile unit goes to seven or eight different shelters during the 2-week rotation and senior students still average nearly 70 surgeries each.

### 2.3. Learning Resources

The intent of the junior experience at shelters is to acquaint the students with the issues animal shelters face and to provide an opportunity to perform several sterilization surgeries. The senior elective is much more in-depth and is designed to:

- Provide veterinary students extensive experience in spaying and castrating animals that will be placed for adoption;
- Provide veterinary students with first-hand experience in dealing with the medical behavioral and surgical problems encountered in animals confined to animal shelters;
- Increase veterinary students’ understanding of the issues surrounding overpopulation of unwanted pets;
- Prepare veterinary students to work closely with local humane societies and animal shelters to find solutions to the overpopulation of unwanted pets in the local community.

**Table 1:** Surgical Instructional Videos. Students review videos of all surgical techniques and procedures prior to performing surgery on the mobile veterinary units.

Adult Dog Castration
Adult Dog Spay
Cat Castration
Cat Spay
Closure
Cryptorchid (Abdominal—Spay Hook technique)
Cryptorchid (Abdominal—under bladder technique)
Cryptorchid (Subcutaneous)
Flank Spay
Millers Knot (Hand tie)
Pedicle Tie
Puppy Castration
Puppy Spay
Square Knot (Hand tie)
Surgeons Knot (Hand tie)
Born to Die

The surgical techniques used are similar to those used in high volume spay/neuter clinics and are consistent with the standards outlined by the Association of Shelter Veterinarians (ASV). (REF) All students who participate in the program are required to view videos of the surgical techniques prior to performing surgery (See table 1).

Understanding of these surgical techniques is then reinforced by the faculty who scrub in to assist the juniors on both days that they perform surgery and for seniors and extern students on the first day of their rotation.

Senior students and externs in the 2-week elective are given assigned reading to increase their understanding of the problems associated with over-population of unwanted dogs and cats, the standards of care in spay/neuter clinics, and standards of care in animal shelters (See Table 2).

**Table 2:** Assigned Reading. Reading assignments in advance of the shelter elective prepare students to understand the issues that animal shelters routinely face.

Guidelines for Standards of Care in Animal Shelters [4]
The Association of Shelter Veterinarians Veterinary Medical Care Guidelines for Spay-Neuter Programs [5]
How Could You [6]
PTS [7]
Letter from a Shelter Manager [8]

### 2.4. Impact of the Spay Neuter Program

The program has an impact both on the veterinary students and on the shelters that participate. Since the inception of the program over 55,000 sterilization surgeries have been performed at local shelters. Since the first mobile unit was acquired in 2007, over 51,000 surgeries have been performed. The adoption rate of the animals that have been sterilized are extremely high, even in shelters that have high euthanasia rates. The program has clearly saved the lives of numerous animals by increasing the adoption rates (in 2013 collectively the euthanasia rate of the shelters we work with was 62%, but the adoption rate of the animals sterilized in our program was 82%). Furthermore, the animals adopted are sterilized preventing future litters of puppies and kittens, and for some shelters reducing the intake numbers over time.

The increased surgical skills and understanding of the problems of pet overpopulation by the veterinary students is, however, the true impact. Students learn high quality high volume spay/neuter techniques in both pediatric and adult dogs and cats.  On average each student performs 67 sterilizations during his/her 2-week rotation, See Table 3). Doing this many surgeries in a 2-week period increases student skill, efficiency and confidence. Allowing students to work through surgical difficulties and complications also builds their confidence.

**Table 3:** Average number of surgeries of each type performed by senior veterinary students.

	kitten neuter	cat neuter	kitten spay	cat spay	puppy neuter	dog neuter	puppy spay	dog spay	Total
Average per student	5	7	9	8	6	10	11	10	67

Veterinary students participating in the shelter elective at Mississippi State University College of Veterinary Medicine gain extensive surgical experience performing spays and castrations of adult and pediatric dogs and cats.

Students are overwhelmingly positive about the experience. Senior students in the 2-week elective consistently rate the experience as one of the most valuable in their veterinary education.

Student evaluations of the shelter elective at Mississippi State University College of Veterinary Medicine are consistently very positive (See Table 4 and Table 5).

**Table 4:** Student evaluations of the shelter elective since acquisition of the second mobile unit.

	Number of Students Responding						
	Strongly Disagree	Disagree	Neutral	Agree	Strongly Agree	Total Number	Mean on 5 pt. score
The rotation met my expectations	2	0	2	22	158	184	4.82
Participating in the Mobile Spay Neuter trips was valuable	2	0	3	12	167	184	4.86
The assigned articles were valuable	2	1	21	53	107	184	4.42
The rotation gave me an understanding of the issues surrounding overpopulation of unwanted pets	2	0	4	32	146	184	4.74
The rotation gave me increased experience in spaying and castrating animals	2	0	3	8	171	184	4.88
I would recommend this elective to others	2	0	2	11	169	184	4.88
The videos of surgical procedures helped prepare me for the course	2	1	4	23	154	184	4.77

**Table 5:** Typical comments from senior veterinary students.

It was a great experience and I appreciate all the patience and great teaching skills.
I loved this rotation. This was a great rotation to allow the students to have more freedom to improve their surgical skills but to have a doctor there to help with any questions/problems we could encounter. I would absolutely recommend this rotation to others.
I was able to improve my surgical skills and better my understanding of shelter management and current issues.
Great rotation and very valuable surgical experience!
I was able to improve my surgical skills and better my understanding of shelter management and current issues.
This is an amazing elective. I was able to learn a lot and refine my skills and efficiency. The doctors and technicians were fantastic to work with. They were very patient and willing to help out a lot in any situation. This is a very valuable elective.
I did appreciate that I was allowed to manage my own surgical complications; that definitely helped improve my surgical competency and confidence level for managing complications.
This was the most valuable rotation that I have participated in during my vet school experience. I have a strong interest in surgery and feel that my surgical skills have greatly improved due to this experience.

Students appreciate the opportunity to gain extensive surgical experience during the shelter elective at the Mississippi State University College of Veterinary Medicine.

Extern students frequently provide feedback as well: “This rotation is extremely valuable to my education. At my school we only get one spay and one neuter prior to graduation, which is why I came here. You guys have made me much more comfortable in surgery. For that I am so grateful.”

With the elective receiving such positive reviews and with the addition of the second mobile unit enrollment in the course has steadily increased over time (See Table 6).

**Table 6:** Student enrollment. Student enrollment in the shelter elective at Mississippi State University College of Veterinary Medicine has increased consistently since the inception of the program in 2007.

Academic year	Number of Students Enrolled
2007 *	6
2007–2008	37
2008–2009	35
2009–2010	41
2010–2011	48
2011–2012	48
2012–2013 **	70
2013–2014	106
2014–2015 ***	92

*: Rotation began spring semester 2007; **: 2nd Mobile Unit put into operation January 2013; ***: At time of submission this number represents only about 80% of the year

## 3. Program Expansion

### 3.1. Shelter Medical Days

In May of 2013, a second component Shelter Medical Days was added to the program expanding the emphasis from spay/neuter and animal wellness to include shelter disease control and biosecurity. Shelter medical days are currently housed within the 6-week Community Veterinary Services junior rotation. Each student spends at least 3 days of the rotation at an animal shelter in the surrounding area, increasing the number of days spent in a shelter from 2 days on the mobile unit to a total of 5 days. Typically, a shelter medical day begins with a biosecurity walk-through of the shelter. In order to encourage students to look critically at breaks in biosecurity, they are given the task of completing a “photo scavenger hunt”. To complete the scavenger hunt they must take pictures of breaks in biosecurity, of good biosecurity practices, and signs of disease. The only rules are that all of the pictures must be taken on the camera belonging to the shelter medicine program and no employee faces can be in the photos. After all of the students have completed their 3 shelter medical days, the pictures are collated and discussed during a grand rounds session. Discussion includes explanations of why the picture was taken as well as what should be done to correct any breaks in biosecurity or prevent any transmission of disease.

Following the walk through at the shelter, students spend the remainder of the shelter medical day performing physicals, basic diagnostic tests and behavior assessments. Students have access to supplies for basic fecal floats, cytology, skin scrapes or any other easily transportable tests. Students also have access to ophthalmoscopes, otoscopes, and stethoscopes. Under the supervision of a faculty member students perform exams, diagnostic tests and make diagnosis and treatment plans for the animals in the care of the shelter.

Shelter Medical Days give all junior veterinary students experience in routine physical examinations and routine diagnostics. Students encounter conditions that are not commonly presented to the veterinary teaching hospital, but are commonly encountered in private veterinary practice. In addition, this program provides students with an increased understanding of disease control, disease prevention and biosecurity in the shelter environment.

### 3.2. Animals in Focus

In 2014, a third arm, Animals in Focus (AIF), was added to the shelter program. AIF is an elementary school program direct at children in socioeconomically disadvantaged areas in Mississippi. In these areas animal neglect and abuse, such as dog fighting, tend to be more prevalent than in some other areas of the state. The intent of this program is to introduce students to the needs of animals, increase the perceived value of animal life, and to use animals as a teaching tool for individual health of both the pet and the children. AIF uses fun and interactive programs to establish relationships with the students and teachers in the schools. An imperative part of the program is using animals (most often dogs) as teachers. The children play games with the animals, learn how to safely interact with animals, and begin to place a higher value on the lives of animals in general. In addition, the hope is that teachers are able to use pets as teaching tools in the classroom. The expectation is that this will increase enthusiasm in the classroom, improve attendance, and generate higher levels of self-confidence in the students.

While AIF is a great program for children in these areas across the state, it is a benefit for the veterinary students as well. Veterinary students in their third year are invited to volunteer for AIF trips. Generally, 4–5 students participate in each trip. These trips are in addition to the junior year spay neuter trips and the shelter medical days. The students lead games for the elementary school students and help them interact comfortably with animals. These trips, while being fun for the vet students, also expose them to community issues that they might not otherwise see. These trips also encourage the students to get involved in their own communities and give them the tools and experience that they need to do that.

## 4. Conclusions

The shelter program, initiated twenty years ago, started very small with an emphasis almost exclusively on spay neuter. In the twenty year history the program has grown both in size and scope. The program now includes eighteen animal shelters/humane groups and incorporates spay/neuter, basic wellness, routine diagnostics, disease management and prevention and biosecurity in the shelter environment. Furthermore, the program is teaching elementary students the value of animal life and basic health principles for animals and people.

The shelter program, while certainly benefiting the animals, the shelters, and the communities that participate, is directed towards increasing the hands-on practical skills of our veterinary graduates. Students obtain extensive experience performing surgery, performing physical examinations, conducting routine diagnostic tests, diagnosing and managing disease conditions all under direct supervision of veterinary faculty.
